# Diagnostic Role of Chromosomal Instability in Melanoma

**DOI:** 10.1155/2012/914267

**Published:** 2012-10-18

**Authors:** Nitika Dabas, Diana M. Byrnes, Ashley M. Rosa, Mark S. Eller, James M. Grichnik

**Affiliations:** Department of Dermatology, Anna Fund Melanoma Program, Sylvester Comprehensive Cancer Center and Interdisciplinary Stem Cell Institute, Miller School of Medicine, University of Miami, Room 912, 1501 NW 10th Avenue, Miami, FL 33136-1012, USA

## Abstract

Early diagnosis gives melanoma patients the best chance for long term survival. However discrimination of an early melanoma from an unusual/atypical benign nevus can represent a significant challenge. There are no current pathological markers to definitively define malignant potential in these indeterminate lesions. Thus, there is a need for improved diagnostic tools. Chromosomal instability (CIN) is a hallmark of cancer and is markedly prevalent in melanoma. Advances in genomics have opened the door for the development of molecular tools to better segregate benign and malignant lesions. This paper focuses on CIN in melanoma and the role of current diagnostic approaches.

## 1. Introduction

The discrimination of an early melanoma from an atypical/unusual benign nevus represents a significant pathological challenge and may result in a misdiagnosis [1–5]. When analyzing ambiguous melanocytic lesions with standard histological and immunohistochemical procedures there is high variability among expert dermatopathologists [1–5]. Due to increases in malpractice suits and looming legal woes, there is significant pressure on physicians to not miss a melanoma. This pressure may lead to melanoma overdiagnosis, increased medical costs, unnecessary surgeries and therapies, and psychological stress for patients [1–7]. Just as overdiagnosis causes problems, underdiagnosis creates the obvious issue of leaving an aggressive cancer untreated. Thus, there is dire need for improved diagnostic methods and capabilities in differentiating benign nevi from melanoma to avoid these problems. Studies have revealed that in contrast to benign nevi, melanomas demonstrate extensive chromosomal instability (CIN) suggesting a potential role in malignant discrimination [8]. These findings have led to work by many groups to characterize malignant cells by the degree of CIN and to the development of techniques to quantitatively and qualitatively measure CIN. Challenges remain and with continued advances in genomics, the field will continue to evolve. Herein, we review the current findings on CIN in melanoma and the role of CIN in diagnostic approaches.

## 2. What Role Does CIN Play in Oncogenesis?

Genomic instability is a hallmark of cancer [9–12]. One specific form of genomic instability is chromosomal instability (CIN). CIN is defined as an increased rate of chromosomal missegregation leading to aneuploidy. While aneuploidy is frequent in cancer, it is also important to point out that it can also occur in benign tissues [13, 14]. While many mechanisms for CIN have been proposed, a full understanding of the processes driving these events and their role in normal and malignant tissue function have yet to be fully elucidated [9–12, 14].

CIN has bidirectional effects on cell growth. While some chromosomal rearrangements are detrimental to cells, leading to their death, others can be advantageous due to deregulation of gene expression, amplification of oncogenes, and deletion of tumor suppressor genes [9]. Studies have shown that the amount of CIN present in a cell is a significant determinant of whether it causes cancer progression or inhibition of growth. While a moderate amount of CIN is sufficient to cause tumorogenesis, large amounts of CIN are lethal to cancer growth [9]. Thus it is likely that tumor cells need sufficient CIN to overcome genetic checkpoints and allow continued evolution but not so much as to damage critical survival pathways [9]. 

## 3. What is CIN's Role in Melanoma?

**Figure 1:**
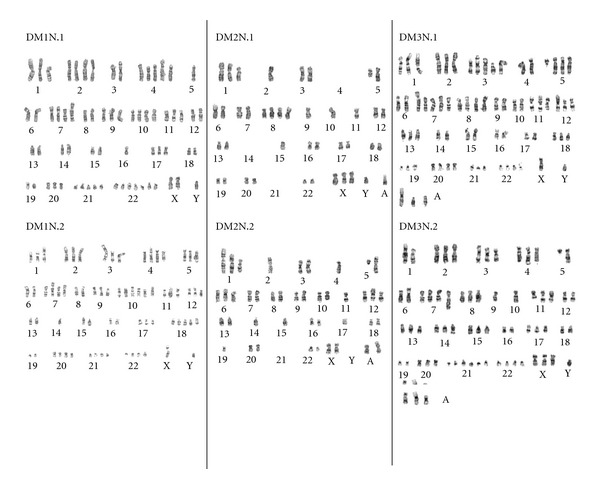
Chromosomal abnormalities are prevalent in melanoma. Shown are pairs of representative karyotypes from 3 melanoma lines, DM1N, DM2N, and DM3N revealing heterogeneity between cells within the culture and overall increases (D1 and D3) and decreases in the total number of chromosomes (D2). Single cell clones from these lines will also expand in culture giving rise to cells with different karyotypes (data not shown) suggesting ongoing CIN.

Studies by Bastian et al. utilized comparative genomic hybridization (CGH) to study chromosomal aberrations in melanoma [8]. CGH uses DNA extracted from the tumor and hybridizes it to a DNA array allowing for detection and fine mapping of amplifications/deletions of genomic DNA segments [15]. CGH revealed that 96% of melanomas exhibited chromosomal aberrations [8]. While it may be inferred that large numbers of chromosomal aberrations are due to ongoing CIN, at a single assay time point it is not possible to define ongoing instability versus a prior event being carried forward in a stable manner. However in melanoma, studies have been conducted to prove that aneuploidy is due to an ongoing CIN process. Studies following melanoma metastases longitudinally suggest that CIN is an ongoing process within each metastasis and that the underlying tumorigenic cell may be more genomically stable than the bulk of the progeny expanding at the metastatic site [16–18]. Genomic aberrations have also been noted in the *in situ* components of tumors suggesting that CIN occurs early in tumorogenesis [19]. Higher variability of genomic aberrations have been noted in metastatic compared to nonmetastatic thin primary tumors suggesting a potential role of CIN in tumor progression [20]. Melanoma also exhibits ongoing CIN in the culture environment (Figure 1). Together these findings suggest CIN plays a critical role in the evolution and progression of melanoma.

## 4. Is There CIN in Benign Neoplasms?

Studies on benign nevi reproducibly demonstrate a lack of significant chromosomal changes with the exception of one type, a nevus called a Spitz nevus [8]. Spitz nevi histologically may be mistaken for melanoma but they are benign lesions. In Bastian's study, 6 of 27 Spitz nevi exhibited amplifications of the entire short arm of chromosome 11, and 1 of 27 Spitz nevi revealed an isolated gain of distal chromosome 7 [8]. The 11p changes amplify DNA that includes the HRAS gene, which has also been found to be specifically mutated in Spitz nevi [21]. The aberrations noted on 11p in Spitz nevi may represent an initiating event that is propagated as the tumor grows. It is possible, unlike melanoma, that there is not significant ongoing instability in these lesions. It is important to point out that others have noted other cytogenetic abnormalities in Spitz nevi [22] so ongoing CIN cannot be fully ruled out. Thus, while the current data suggests CIN does not play a role in the majority of nevi, chromosomal aberrations do occur in benign lesions and indicate a need to be cautious when interpreting chromosomal changes in melanocytic lesions.

## 5. Is CIN Variable Depending on the Type of Melanoma?

Melanoma includes a spectrum of malignant neoplasms including superficial spreading melanoma (SSM) (included with nonchronic sun exposed), lentigo maligna melanoma (LMM) (included with chronic sun exposure), acral lentiginous melanoma (ALM), and mucosal melanoma. Studies utilizing CGH have noted marked abnormalities of chromosomes in melanoma (Table 1) [8, 23, 24]. The most frequently gained regions in melanoma were 1q, 6p, 7p, 7q, 8q, 17q, and 20q while the most frequent losses were seen at 6q, 9p, 9q, 10p, 10q, and 11q [8]. Interestingly, some chromosomal aberrations and the extent of aberrations appear to differ among melanoma subtypes [23] suggesting differences in CIN pathways.

More recently, Curtin et al. found alterations (mutations and amplifications) in the 14q12 locus in some melanomas from all groups except the SSM/nonchronic sun exposure group [24]. Examination of this region revealed amplifications and mutations of KIT, a gene critically involved in the homeostatic pathways of human cutaneous melanocytes [25]. The presence of specific genomic changes in different melanoma subtypes provides further evidence pointing to inherent differences in their genomic evolution. At this point it is not entirely clear if the state of cellular differentiation, the type of underlying mutations or the local environment in which the tumor develops has the greatest impact on the differences seen in the chromosomal changes. A greater understanding of this process may provide valuable insight into the nuclear structural and genetic changes that occur as melanoma develops.

Another interesting finding is the loss of 9p21 (including tumor suppressor genes CDKN2A, CDKN2B, and ARF) in 56% of common and 54% of dysplastic nevi that are associated with melanoma [26]. Loss of 9p21 was not seen in control nevi not associated with melanoma. This suggests that loss of 9p21 may be a cytogenetic marker for nevi with high potential to progress to melanoma [26].

Thus these findings suggest that the patterns of chromosomal aberrations in melanocytic lesions have potential diagnostic and prognostic value.

## 6. What Techniques Can We Utilize to Diagnose Genomic Instability?

**Figure 2:**
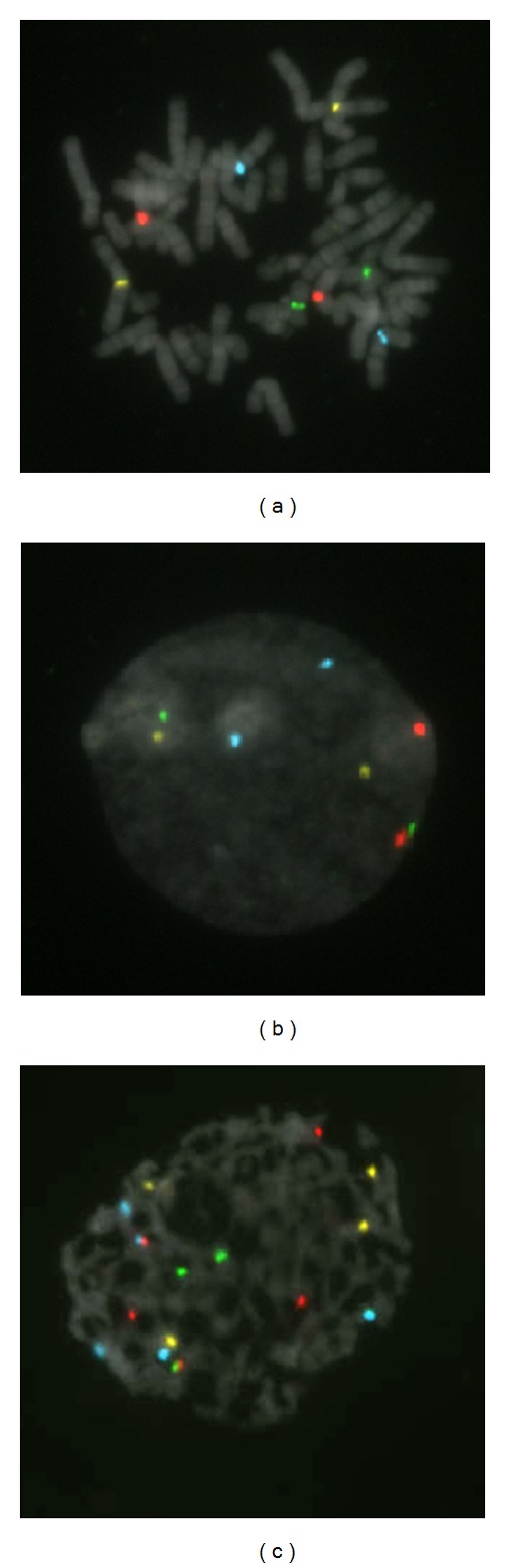
FISH utilizes fluorescent probes to specific areas on chromosomes in cultured cells. Illustrated are OligoFISH probes to the centromeres of chromosomes 2 (yellow), 6 (light blue), 7 (green), and 8 (red). (a) demonstrates the binding to specific chromosomes in a metaphase spread; (b), binding to DNA sequences in a normal diploid human cell, and (c), binding to sequences in a cell with an abnormally increased chromosomal number.

Currently, two major assay methods are being used to detect CIN in melanomas. One approach, noted above, is CGH. The advantage of CGH is the fine detail to which genomic changes can be mapped. However, this approach can be costly and because the DNA is pooled, the changes detected represent an average and do not provide data on the genetic heterogeneity that may exist between tumor cells. A second commonly applied approach is Fluorescence *in situ* Hybridization (FISH) [27, 28]. FISH allows the detection of copy number changes on chromosomal regions using DNA probes directly on individual cells. The DNA probes may target chromosomal specific centromeric regions as shown in cells in culture (Figure 2), or may be targeted to bind to specific gene loci. One of the disadvantages of FISH is that only a limited number of probes can be imaged at a time. However, its relatively low cost, the ability to perform analysis on tissue sections, ability to examine individual cells and use with automatic imaging systems is increasing its diagnostic use [29]. As genomic technologies move forward there will certainly be improved technologies.

## 7. How Well Has FISH Done? 

Given the advantages of FISH, most efforts are focusing on its use for melanoma diagnosis (Table 2) [6, 30–36]. FISH probes may be designed to identify chromosomes or specific gene loci. Utilization of probes specific to chromosomes 6, 7, 11, and 20 (Cellay, Cambridge, MA) revealed significant changes between benign nevi and melanoma [30]. Chromosomal abnormalities were noted in only 2 of the 32 benign nevi while 29 of the 31 melanomas revealed changes (*P* < 0.0001). Overall sensitivity and specificity were both reported as 94%. Many studies have been performed with a 4 probe set (Vysis/Abbott Molecular, Des Plaines, IL) that targets 6p25: ras responsive element binding protein 1 (RREB1), 6q23: v-myb myeloblastosis viral oncogene homologue (MYB), CEP6 (centromere 6), and 11q13: cyclin D1 (CCND1) [29]. One study showed the sensitivity and specificity of 100% in discriminating between nodular melanoma and mitotically active nevi, though the study was conducted on a small sample of ten cases [37]. Table 2 depicts the sensitivity and specificity of five FISH studies done with the above 4 probe set [6, 31–34]. 

Although the results of the first four studies with the probe set revealed FISH to be a relatively reliable tool in diagnosing melanoma properly, a study by Gaiser et al. in 2010 was not as encouraging [34]. In this study they looked at the correlation of FISH with patient outcome. The results did not achieve clinically useful sensitivity or specificity. However, CGH did reveal significantly more chromosomal aberrations in the melanocytic lesions that developed metastasis.

The ability of FISH to determine ploidy of cells has also been shown in a study conducted by Satoh et al. in 2000 [36]. Using alpha-satellite DNA probes, D18Z1, DXZ1, and DYZ3, they were able to detect tetraploidy in all of their melanomas. However, tetraploidy can also be identified in Spitz nevus samples and may need to be controlled for [31]. A recent study by Gerami et al. with a new probe set including CDKN2A (9p21), RREB1 (6p25), MYC (8q24), and CCND1 (11q13) that allows also for better control of tetraploidy, revealed a sensitivity of 94% and specificity of 98% for melanoma; thus, demonstrating a marked improvement in diagnostic value [35]. It is of some interest that a tetraploid state has been shown to precede aneuploidy and acquisition of malignant behavior in prostate, breast, and ovarian cancer models [38, 39].

FISH has also been used for uveal melanoma. Van den Bosch et al. used probes for chromosomes 1, 3, 6, and 8 and correlated alterations in the number of these chromosomes with survival of patients with uveal melanoma [40]. They identified statistically significant relationships between certain alterations of the numbers of these chromosomes with survival, with monosomy 3 (*P* = 0.002), and gain of chromosome 8 (*P* = 0.002) as the most significant genetic changes detected that correlate with poor prognosis. Similarly, Patel et al. examined loss or gains of chromosomes 3 and 8 in 33 uveal melanomas [41]. Sixteen (48%) of those tumors were found to have genetic imbalances and 14 (88%) of those patients had died by the end of the study. Of the 51% that showed no genetic imbalances, only 5 patients (29%) had died by the end of the study. 

In summary, FISH is a promising technique and segregates well with clearly benign or malignant lesions. However, indeterminate melanocytic lesions still pose a challenge. Given recent probe improvements and data on uveal melanoma, FISH is likely to have an increasing role in prognosis.

## 8. Where Else Can We Take This in the Future?

Rapid advances in technology may allow for numerous different color probes/channels to be analyzed simultaneously thus allowing for a more extensive determination of chromosome number and specific gene amplifications or deletions. Further it is possible that with increased sensitivity, mutation specific probes could also be created and utilized. Ultimately these approaches will allow for a more thorough analysis of CIN and specific defects in molecular pathways on a cell by cell basis.

It is possible that these approaches will eventually surpass standard histopathologic diagnosis in the determination of which lesions are most likely to be lethal. Further, these techniques may also be used to characterize the different subsets of melanomas and may be used to determine the optimal drugs for treatment.

## 9. Conclusion

Melanoma is an extremely aggressive and deadly form of cancer. Early detection and diagnosis remains the best way to save lives from this disease. However, early melanoma can be difficult to distinguish from unusual/atypical nevi and every year the number of melanoma diagnoses increases [42, 43]. In contrast to benign nevi, melanoma demonstrates marked CIN. The pathways involved in the process still need to be fully defined. Nevertheless, current technologies detecting differences in CIN have diagnostic value and as the technologies continue to improve, they have the potential to eventually surpass the accuracy of standard histopathologic diagnosis.

## Figures and Tables

**Table 1:** Common chromosomal aberrations found in specific melanoma subtypes [8, 23, 24].

Subtype of melanoma	Common chromosomal aberrations		Statistically significant chromosomal aberrations	
	Gains	Losses	Gains	Losses
Superficial spreading, nonchronic sun exposure	6p, 7, 8q, 17q, 20q [23]	10q22.1, 10pter [8], 9p, 10q, 21q [23]		10q* [23]

Lentigo maligna, chronic sun exposure	17pter, 15q21.1, 15q15 [8], 6p, 11q13, 17q, 20q [23], 4q12 [24]	13q21.1, 17qter, 17pter [8], 6q, 8p, 9p, 13, 21q [23]		

Acral	12q14, 5pter, 11q13, 4q11 [8], 6p, 7, 8q, 17q, 20q, 5p15, 5p13, 11q13, 12q14 [23], 4q12 [24]	15q13, 16q24, 16q23.1 [8], 6q, 9p, 10q, 11q, 21q [23]	12q14, 5pter [8], 69, 11q13 [23]	10q* [23]

Mucosal	1q, 6p, 7, 8q, 11q13, 17q, 20q, 1q31, 4q12, 12q14 [23], 4q12 [24]	3q, 4q, 6q, 8p, 9p, 10q, 11p, 11q, 21q [23]	1q, 6p, 11q13, 17q, 12q14 [23]	3q, 8p, 10q*, 11p [23]

*The losses in 10q were significant compared to melanomas from chronic sun exposed skin.

**Table 2:** The sensitivity and specificity of recent studies using FISH assays to identify melanoma.

Probe set used	Research study	Sensitivity %	Specificity %	Melanoma tested (+/total)	Typical nevi tested (+/total)	Ambiguous lesion tested	Number of experts reviewing results
chromosome 6, 7, 11, and 20	Hossain et al. [30]	94%	94%	29/31	2/32	0	2

6p25 (RREB1) 6q23 (MYB) CEP6 11q13 (CCND1)	Gerami et al. [6]	86.7	95.4	72/83	4/86	12/27	2
	Fang et al. [31]	82	98	41/50	1/50*	0	**
	Vergier et al. [32]	85	90	17/20	2/19	23/90	3
	Abásoloet al. [33]	100	94.1	27/27	1/9	1/9	2
	Gaiser et al. [34]	50***	60***	7	3	12	3

9p21 (CDKN2A)							
6p25 (RREB1) 11q13 (CCND1)	Gerami et al. [35]	94	98	**/51	**/51	0	**
8q24 (MYC)							

D18Z1							
DXZ1	Satoh et al. [36]	100	100	8/8	0/8	0	**
DYZ3							

*Corrected for tetraploidy.

**Exact number not specified.

***Based on clinical behavior.
